# Identification of novel hypermethylated genes and demethylating effect of vincristine in colorectal cancer

**DOI:** 10.1186/1756-9966-33-4

**Published:** 2014-01-06

**Authors:** Ji Wook Moon, Soo Kyung Lee, Jung Ok Lee, NamI Kim, Yong Woo Lee, Su Jin Kim, Ho Jin Kang, Jin Kim, Hyeon Soo Kim, Sun-Hwa Park

**Affiliations:** 1 Department of Anatomy, Institute of Human Genetics, Korea University College of Medicine, 126-1, Anam-dong 5-ga, Seongbuk-gu, Seoul 136-705, Republic of Korea; 2 Department of General Surgery, Korea University Medical Center, 126-1, Anam-dong 5-ga, Seongbuk-gu, Seoul 136-705, Republic of Korea

**Keywords:** Colorectal cancer, Hypermethylated genes, Therapeutic targets, CIMP markers, 5-aza-2′-deoxycytidine, Vincristine

## Abstract

**Background:**

Colorectal cancer (CRC) arises as a consequence of genetic events such as gene mutation and epigenetic alteration. The aim of this study was to identify new hypermethylated candidate genes and methylation-based therapeutic targets using vincristine in CRC.

**Methods:**

We analyzed the methylation status of 27,578 CpG sites spanning more than 14,000 genes in CRC tissues compared with adjacent normal tissues and normal colon tissues using Illumina bead chip array. Twenty-one hypermethylated genes and 18 CpG island methylator phenotype markers were selected as candidate genes. The methylation status of 39 genes was validated by quantitative methylation-specific polymerase chain reaction in CRC tissues, adjacent normal tissues, normal colon cells, and three CRC cell lines. Of these, 29 hypermethylated candidate genes were investigated using the demethylating effects of 5-aza-2′-deoxycytidine (5-aza-dC) and vincristine in CRC cells.

**Results:**

Thirty-two out of 39 genes were hypermethylated in CRC tissues compared with adjacent normal tissues. Vincristine induced demethylation of methylated genes in CRC cells to the same extent as 5-aza-dC. The mRNA expression of *AKR1B1*, *CHST10*, *ELOVL4*, *FLI1*, *SOX5*, *STK33*, and *ZNF304* was restored by treatment with 5-aza-dC and vincristine.

**Conclusion:**

These results suggest that these novel hypermethylated genes *AKR1B1*, *CHST10*, *ELOVL4*, *SOX5*, *STK33*, and *ZNF304* may be potential methylation biomarkers and therapeutic targets of vincristine in CRC.

## Background

Colorectal cancer (CRC) is the third most common cancer and the second leading cause of cancer death in the world. CRC is a consequence of genetic events including gene mutations and epigenetic alterations that transform colonic epithelial cells into adenocarcinoma cells [1]. The early detection of CRC is most important in cancer patients to reduce cancer mortality [2]. Different stages of CRC have different prognoses and the effects of adjuvant chemotherapy differ between CRC stage II and stage III [3]. Current CRC chemotherapy consists of a combination of cytotoxic DNA antimetabolites, such as 5-fluorouracil, leucovorin, or oxaliplatin. However, the best combination of these anticancer drugs is still not fully established [4]. To achieve this, epigenetic DNA methylation was reported as a suitable approach for a better understanding of CRC progression and therapeutic targets [5].

A great number of studies have focused on the epigenetic alterations of tumor suppressor genes in the regulation of cancer initiation and progression [6]. Gene-specific methylation changes in promoter CpG regions have been largely related to biological processes of tumor progression including cell proliferation, communication, adhesion, mobility, signal transduction, and drug resistance [7]. Aberrant methylation of CpG islands in the promoter or exon 1 regions of tumor suppressor genes has been correlated with transcriptional silencing of downstream genes in colorectal cancer [8,9]. Many genes silenced by aberrant methylation, including *CDKN2A*, *THBS*, and *SFRP*, have been proposed to be associated with CRC tumorigenesis [10-13]. Moreover, promoter methylation was also referred to as the CpG island methylator phenotype (CIMP) [14,15]. CIMP-positive CRC was distinguished from CIMP-negative CRC patients by clinicopathological factors [16], and CIMP was associated with development of the serrated pathway of CRC [17]. Clinically, several CIMPs containing *MLH1* (a mismatch repair gene), and microsatellite instability were characterized to be associated with CRC prognosis [18,19]. Furthermore, a panel of CIMP including *RUNX3*, *CACNA1G*, *IGF2*, and *MLH1* consists of specific markers for clinical trials [20]. Hughes et al. reviewed the existing literature of 640 potential relevant papers to summarize CIMPs in CRC [21]. Although there are many lines of evidence that have been proposed as potential biomarkers for CRC in humans, many researchers continue to research new CRC-specific methylation markers. Recently, methylation chip array techniques have been widely used to identify new DNA methylation biomarkers in CRC. However, array data are needed to confirm other methods such as quantitative methylation polymerase chain reaction (PCR) (QMSP), methylation-sensitive high-resolution melting, and pyrosequencing [22-24]. QMSP is a sensitive tool and offers quantitative analysis of DNA methylation status [25].

Vincristine is a vinca alkaloid from the plant *Catharanthus roseus*, and mainly arrests mitosis in metaphase by binding to tubulin dimers [26]. It is used as a chemotherapy drug for various types of cancers, including non-Hodgkin’s lymphoma [27], acute lymphoblastic leukemia, lung cancer, breast cancer, and CRC [28-31]. Recently, cyclophosphamide, vincristine, and prednisone (COP) chemotherapy was used to significantly improve overall survival and progression-free survival in primary colonic lymphoma patients [32]. There was one report that low concentration of vincristine reduced the methylated cytosine in human lung adenocarcinoma cells [33]. However, the DNA methylating-based effects of vincristine are still unknown for methylation marker genes in CRC.

In this study, to identify new hypermethylated candidate genes in CRC patients, we analyzed methylation profiles using bead chip array-based technology and QMSP. In addition, to identify methylation-based therapeutic target genes, the demethylating effect of vincristine was examined using 21 hypermethylated candidate genes and 18 CIMP markers. Correlations between methylation status and mRNA expression were analyzed by reverse-transcription PCR.

## Methods

### Tissues

Thirty-one pairs of colorectal cancer (CRC) tissues and adjacent normal tissues and 10 normal colon tissues were obtained from the Department of Colorectal Surgery, Korea University Medical Center. The characteristics of each subject are summarized in Table 1. This study was approved by the institutional review board of Korea University and informed consent was obtained (IRB No. KU-IRB-10-08-A-1). The diagnosis of CRC tissues was acquired from pathology reports, the institutional review board, and histological evaluations. Fresh tissue samples were frozen in liquid nitrogen after resection and stored at −80°C.

**Table 1:** Clinicopathologic characteristics of colorectal cancer patients

**Characteristics**	**Methylation-profiling subjects**		**Validation subjects**
	**Non-patients (n = 10)**	**Patients (n = 21)**	**Patients (n = 10)**
Age (years)			
> 65	4	13	5
≤ 65	6	8	5
Gender			
Male	2	14	5
Female	8	7	5
Location			
Rectum		5	5
Right, left colon	10	16	5
Clinical stage			
I + II		11	5
III + IV		10	5
Differentiation			
Well		7	5
Moderate		14	5
Lymphatic metastasis			
Present		9	5
Absent		12	5
Maximum tumor diameter (mm)			
> 25		7	5
≤ 25		14	5
Smoking (1 pack/day)			
Yes		9	4
No		12	6
Alcohol intake (3 times/week)			
Yes		11	4
No		10	6

### Cell lines

One normal colon cell line (CCD18Co) and three CRC cell lines (SW480, Dukes’ type B; DLD-1, Dukes’ type C; LoVo, Dukes’ type C and stage IV) were obtained from the American Type Culture Collection (Manassas, VA, USA). CCD18Co cells were cultured in Eagle’s minimum essential medium and the three CRC cells were cultured in RPMI 1640 medium, all supplemented with 10% fetal bovine serum (Hyclone, Logan, UT, USA) and 1% penicillin/streptomycin (P/S; Invitrogen), and maintained at 37°C and 5% CO_2_ atmosphere.

### Genomic DNA extraction

Genomic DNA was extracted using the QIAamp DNA Mini-Kit (Qiagen, Valencia, CA, USA) according to the manufacturer’s recommendations. Tissue samples were ground up by 3-mm diameter punches and then mixed with 700 μL lysis buffer containing 20 μg/mL Labo Pass protease K (Cosmo Gene Tech., Seoul, Korea), 20 mM Tris∙HCl (pH 8.0), 5 mM EDTA (pH 8.0), 400 mM NaCl, and 1% SDS solution (Sigma-Aldrich, St. Louis, MO, USA). The mixed samples were incubated at 42°C overnight. After incubation, genomic DNA was purified by phenol/chloroform extraction. Genomic DNA was eluted in 100 μL of water and quantified with a NanoDrop ND-100 device (Thermo Fisher Scientific, Hudson, NH, USA).

### Sodium bisulfite DNA modification

Two micrograms of genomic DNA in a volume of 20 μL RNase-free water was bisulfite-converted using the EpiTect fast DNA bisulfite kit (Qiagen). Bisulfite conversion was performed according to the manufacturer’s recommendations. The reaction was performed by mixing 85 μL bisulfite mix solution and 35 μL DNA protect buffer in 200 μL PCR tubes at room temperature. The bisulfite-converted genomic DNA was eluted from the column with 100 μL dH_2_O and stored at −80°C until use.

### Methylation bead chip array

Human Methylation 27 DNA Analysis Bead Chip (Illumina Inc., San Diego, CA, USA) is a methylation-profiling technology based on bisulfite modification of DNA. This bead chip array can provide methylation information at a single-base resolution for 27,578 CpG sites spanning more than 14,000 genes. One microgram of bisulfite-converted genomic DNA was applied to the bead chips using Illumina-supplied reagents and conditions. After extension, the array was fluorescently stained and scanned, and the intensities of the M (methylated) and U (unmethylated) bead types were measured. Each methylation data point is represented by fluorescent signals from the M and U alleles. The ratio of fluorescent signals was then computed from the two alleles; *β* value = (max (M, 0))/(|U| + |M| + 100). The *β* value reflects the methylation level of each CpG site. A *β* value of 0–1.0 indicates the percent methylation from 0% to 100%, respectively.

### Quantitative methylation-specific PCR (QMSP)

Quantitative methylation status in the bisulfite-converted genomic DNA was confirmed by quantitative real-time PCR using the 7000 HT Real-Time PCR System (Applied Biosystems) according to the manufacturer’s recommendations. Methylation primers for 21 candidate genes and 18 CIMP markers were designed using the MethPrimer software (http://www.urogene.org/methprimer/). Primers for QMSP were designed for large promoter CpG islands containing detected CpG sites near the transcription start site (Additional file 1: Table S1). PCR reactions were performed using an optical 96-well tray in a final volume of 20 μL. The reaction mixture consisted of 5 μL 2X Maxima SYBR Green/ROX qPCR master mix (Thermo Fisher Scientific), 250 nM of each primer, and 30 ng of bisulfite-converted DNA template. The QMSP program was as follows: 50°C for 2 min and 95°C for 10 min, followed by 45 cycles at 95°C for 15 s, and then 60°C for 1 min. After PCR, a thermal melt profile was performed to examine the homogeneity of the PCR application. Each DNA sample was analyzed in duplicate, and the mean quantity was used for further analysis. Relative quantification of the amplified gene levels in the bisulfite-converted genomic DNA sample was performed by measuring the threshold cycle (C_T_) values of target genes and β-actin (*ACTB*). The mean quantity of genes was divided by the mean quantity of *ACTB* and was used for the normalization of input DNA. The negative values for *ACTB* were excluded from the methylation analysis. The bisulfite-converted genomic DNA of a known concentration was drawn at 1, 1/4, 1/16, and 1/64 via serial dilutions, and then used in a standard curve for quantification. The modified genomic DNA by CpG methyltransferase M.SssI (NEB, Ipswich, MA, USA) was used as a positive control according to the manufacturer’s recommendations. DNA methylation according to M.SssI was verified using the restriction enzyme BstUI (NEB).

### Reverse-transcription PCR

mRNA was extracted using the commercial RNeasy Mini-kit (Qiagen, Hilden, Germany) according to the manufacturer’s recommendations. The mRNA was eluted in 20 μL of DEPC water (Qiagen) and quantified with a NanoDrop ND-100 device (Thermo Fisher Scientific). One microgram of mRNA from each sample was subjected to cDNA synthesis using Maloney murine leukemia virus RT and random hexamers (Promega, Madison, WI, USA). cDNA synthesis was performed according to the manufacturer’s recommendations by mixing 1 μL of 1 μg mRNA, 4 μL 5X RT buffer, 1 μL 500 nM oligo dT, 1 μL 10 mM dNTP, 0.5 μL RNasein, 1 μL M-MLV reverse transcriptase, and 11.5 μL dH_2_O in PCR tubes. The mixture was then incubated at 37°C for 1 h. cDNA was diluted with 20 μL dH_2_O and stored at −80°C until use. Primers were designed using primer3 version 0.4.0 (http://primer3.ut.ee/) and are shown in Additional file 1: Table S2. cDNA was amplified by PCR with primers for each target gene. The RT-PCR program was as follows: 95°C for 10 min, followed by 35 cycles at 95°C for 15 s, 60°C for 15 s, and then at 72°C for 45 s. *ACTB* was amplified simultaneously with the other PCR products and was used as a control for RNA integrity.

### Chemical treatment

To determine the optimal concentration of 5-aza-2′-deoxycytidine (5-aza-dC) and vincristine (Sigma-Aldrich) in CRC cell lines, we measured cell viability with the MTT assay (Additional file 2: Figure S1) according to the manufacturer’s recommendations using MTT reagents (10 μL/well, 7.5 mg/mL in phosphate-buffered saline) and dimethyl sulfoxide (50 μL/well, Sigma-Aldrich). To identify the demethylating effect of treatment with anticancer drugs, CCD18Co, SW480, DLD-1, and LoVo cells were seeded in six-well culture plates (SPL LifeSscience, Pocheon, Korea) at a density of 0.5 × 10^5^ cells per well. After 24 h, cells were cultured in serum-free media containing either 30 μM 5-aza-dC or 100 nM vincristine in 10 μL dimethyl sulfoxide for 48 h at 37°C in a 5% CO_2_ atmosphere. After 48 h, cells were washed in PBS (Sigma-Aldrich) three times and then harvested.

### Statistical analysis

The statistical significance of the methylation bead chip array data was determined using a paired t-test based on Δ*β* means (means of *β* value in CRC tissues – means of *β* value in normal tissues). The methylated intensity ratio in CRC was confirmed by fold-change (methylated intensity ratio of CRC tissues/methylated intensity ratio of normal colon tissues) and odds ratio (methylated intensity ratio contract of unmethylated intensity in CRC). The false discovery rate (FDR) was controlled by adjusting the *P* value using the Benjamini-Hochberg algorithm, the incorrectly substituted probabilities of specifically observed methylation CpG sites for each gene in *P* values. The methylated intensity ratio of QMSP was determined by the percentage of methylated reference (PMR) gene, and the PMR value was defined as [(GENE)_sample_/(ACTB)_sample_]/[(GENE)_M.SssI_/(ACTB)_M.SssI_] × 100. The significance of different PMR values among CRC tissues and adjacent normal tissues was defined with the chi-squared test and analysis of variance test using Sigma Stat (SPSS Inc., Chicago, IL, USA). All statistical tests were two-sided and *P* values of *<* 0.05 were considered to indicate statistical significance.

## Results

### Selection of 21 hypermethylated candidate genes in CRC

**Figure 1:**
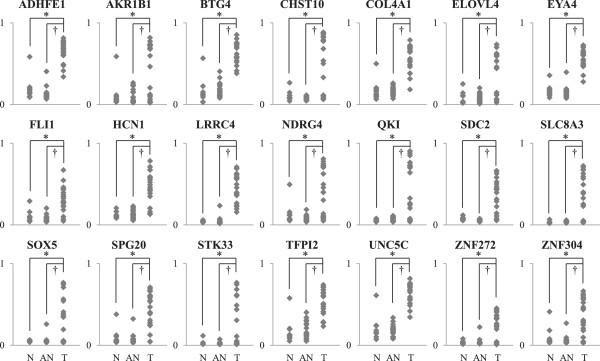
**Hypermethylation of promoter CpG sites for 21 genes by methylation chip array.** The methylation statuses of CpG sites in promoter CpG islands for 21 genes were identified using methylation chip array in 10 normal colon tissues, 21 CRC tissues, and 21 adjacent normal tissues. All genes were significantly strongly hypermethylated in their promoter CpG islands in CRC tissues compared with adjacent normal tissues and normal tissues (*P* < 0.05). N: normal colon tissues, T: CRC tissues, AN: adjacent normal tissues.

To identify the aberrant methylation of various genes in CRC, we performed a methylation chip array in 10 normal colon tissues, and 21 CRC tissues and adjacent normal tissues. We found a total of 3,177 CpG sites in the promoter regions (1,704 CpG sites, 53.6%) and non-promoter regions (1,473 CpG sites, 46.4%), with aberrant methylated CpG sites identified in CRC tissues compared with adjacent normal and normal colon tissues, according to statistical significance determined by the paired t-test and an FDR *P* value of <0.001 based on a Δ*β* mean of 0.1. Among 3,177 CpG sites, we identified 597 genes with hypermethylated CpG sites in promoter CpG islands (data not shown). Finally, we selected 21 candidate genes that contained strongly hypermethylated CpG sites in promoter CpG islands in CRC tissues compared with adjacent normal tissues (Figure 1).

### Validation of 39 genes by QMSP

**Figure 2:**
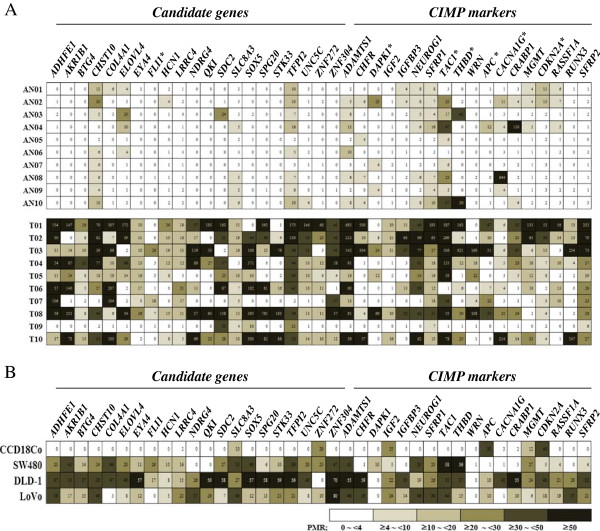
**Validation of methylation status of 21 candidate genes and 18 CIMP markers.** The methylation status of 21 candidate genes and 18 CIMP markers was validated using QMSP in 10 CRC tissues compared with adjacent normal tissues **(A)**, normal colon cells, and three CRC cell lines **(B)**. Methylated intensity ratio of QMSP data was determined by the PMR values. The significance of different methylation statuses in CRC tissues and adjacent normal tissues was defined with the analysis of variance test using Sigma Stat. Values of *P* < 0.05 were considered to indicate statistical significance. * indicates the methylation status of genes in CRC compared with adjacent normal tissues was not statistically significant.

To confirm the methylation status of 21 candidate genes from the array results and 18 CIMP markers, we validated the methylation status in the promoter CpG islands of selected genes by QMSP in 10 different CRC tissues compared with adjacent normal tissue. The quantitative analysis with the PMR value supported the differential methylation status between CRC and normal tissues. The methylation status in the promoter CpG islands of all candidate genes was frequently higher in CRC tissues compared with adjacent normal tissues except *FLI* (Figure 2A). The methylation status of 12 CIMP markers, namely, *ADAMTS1*, *CHFR*, *IGF2*, *IGFBP3*, *NEUROG1*, *SFRP1*, *WRN*, *CRABP1*, *MGMT*, *RASSF1A*, *RUNX3*, and *SFRP2* was also frequently higher in CRC tissues compared with adjacent normal tissues (Figure 2A). In normal colon cells, *SLC8A3*, *ZNF272*, *IGF2*, *APC*, *MGMT*, and *CDKN2A* were methylated. All genes were hypermethylated in one or more CRC cell lines except *WRN* (Figure 2B).

### Demethylation effect of vincristine on 29 hypermethylated genes in CRC cell lines

**Figure 3:**
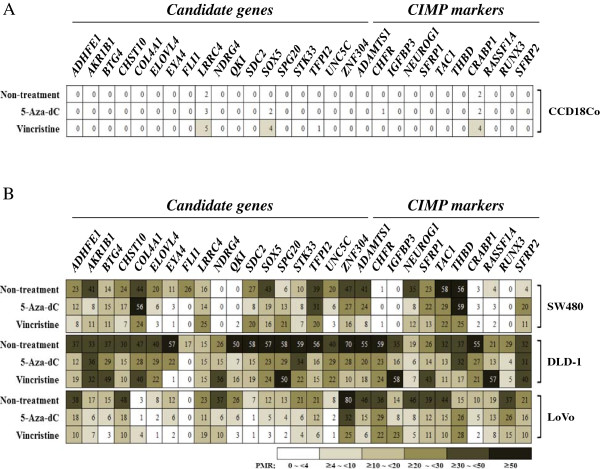
**Demethylating effect of 5-aza-dC and vincristine on methylated genes in three CRC cell lines.** Changes in the methylation status of hypermethylated genes in normal colon cells **(A)** and CRC cells **(B)** were identified by QMSP after treatment with 30 μM 5-aza-dC or 100 nM vincristine for 48 h at 37°C. Methylated intensity ratio was determined by the percentage of methylated reference (PMR).

The 10 genes hypermethylated in normal colon cells or not significantly hypermethylated in tumor tissue were excluded for chemical treatment. Eighteen candidate genes and 11 CIMP markers were selected to identify the demethylating effects of vincristine. The methylation status of 29 genes was determined by PMR values. In normal colon cells, most genes were not affected by 5-aza-dC and vincristine treatment (Figure 3A). In contrast, 14 candidate genes and seven CIMP markers were significantly demethylated by 5-aza-dC treatment in two CRC cell lines (Figure 3B). In addition, 12 candidate genes (*ADHFE1*, *AKR1B1*, *CHST10*, *ELOVL4*, *EYA4*, *FLI1*, *QKI*, *STK33*, *SOX5*, *UNC5C*, and *ZNF304*) and eight CIMP markers (*ADAMTS1*, *CHFR*, *CRABP1*, *NEUROG1*, *RUNX3*, *SFRP1*, *THBD*, and *TAC1*) were significantly demethylated by vincristine treatment in two more CRC cell lines (Figure 3B).

### Restoration of mRNA expression by vincristine in DLD-1 cells

**Figure 4:**
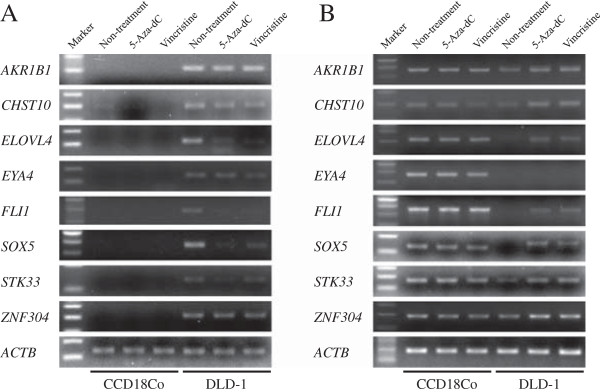
**Association between DNA methylation and mRNA expression of eight genes in CCD18Co and DLD-1.** The DNA methylation status **(A)** and mRNA expression **(B)** of eight genes, including six newly identified hypermethylated genes, was measured using QMSP and RT-PCR in CCD18Co and DLD-1 cells following 30 μM 5-aza-dC or 100 nM vincristine treatment for 48 h at 37°C. *ACTB* was amplified simultaneously with the other PCR products and was used as a control for DNA methylation and mRNA integrity. Marker: molecular weight DNA marker.

The effect of methylation on mRNA expression was investigated by MSP and RT-PCR analysis in 5-aza-dC- and vincristine-treated DLD-1 and CCD18Co cells. The methylation status of *CHST10*, *ELOVL4*, *EYA4*, *FLI1*, *STK33*, *SOX5*, and *ZNF304* was decreased by treatment with 5-aza-dC and vincristine in DLD-1 cells, but were not changed in CCD18Co cells. The methylation status of *CHST10*, *ELOVL4*, *EYA4*, and *ZNF304* was highly decreased by vincristine (Figure 4A). The mRNA expression of *AKR1B1*, *CHST10*, *ELOVL4*, *FLI1*, *STK33*, *SOX5*, and *ZNF304* was increased by treatment with 5-aza-dC and vincristine in DLD-1 cells, but *EYA4* mRNA expression was not detected. The mRNA expression levels of all genes were not affected by 5-aza-dC treatment in CCD18Co cells (Figure 4B). The methylation of *AKR1B1* was not decreased significantly by treatment with 5-aza-dC or vincristine, but the mRNA expression levels of this gene were increased (Figure 4). These results suggest that vincristine promotes the demethylation of *CHST10*, *ELOVL4*, *FLI1*, *SOX5*, *STK33*, and *ZNF304*, and the methylation-mediated silencing or down expression of these genes was restored by vincristine in DLD-1 cells to the same extent as 5-aza-dC, as measured by mRNA expression.

## Discussion

This study identified novel hypermethylated genes in CRC through a genome-wide study. DNA hypermethylation leads to the downregulation and silencing of tumor suppressor genes during the pathogenesis of various human cancers [10,34-36]. Recently, genome-wide array-based studies have reported altered DNA methylation gene profiles in CRC [22-24]. Oster et al. identified hypermethylated *FLI1*, *ST6GALNAC5*, *TWIST1*, *ADHFE1*, *JAM2*, *IRF4*, *CNRIP1*, *NRG1*, and *EYA4* genes in the adenomas and carcinomas of colorectal lesions [22]. Kim et al. also reported 20 top-ranking hypermethylated genes in CRC [23]. Mori et al. identified several novel candidate CRC biomarkers including *VSX2*, *BEND4*, *NPTX1*, *BTG4,* and *GLP1R*[24]. In our methylation chip array results, we discovered 1,411 hypermethylation CpG sites that were located in the promoter CpG islands of 597 genes (data not shown), and selected 21 candidate genes for further study (Figure 1). Several candidate genes were consistent with previous reports, such as *BTG4*, *FLI1*, *TWIST1*, *ADHFE1*, *UNC5C*, and *SPG20*[22-24]. We validated the methylation status in the promoter CpG islands of candidate genes by QMSP for the investigation of large contiguous CpG sites, the results of which were concordant with the array results for most genes except *FLI1* (Figure 2A).

Among the various CIMP markers in CRC, 18 CIMP markers were selected for the validation of methylation status and methylation-based therapeutic targets in CRC. *ADAMTS1*, *CHFR*, *DAPK1*, *IGF2*, *IGFBP3*, *NEUROG1*, *SFRP1*, *TAC1*, *THBD*, and *WRN* were also hypermethylated in our chip data (data not shown). In our QMSP results of CIMP markers, *DAPK1*, *TAC1*, *THBD*, *APC*, *CACNA1G*, and *CDKN2A* were not significantly methylated in CRC tissues (Figure 2A). These discrepancies may be due to differences in the epigenomes of tumors or patient ethnic background.

The hypermethylation of *AKR1B1*, *CHST10*, *ELOVL4, SOX5*, *STK33*, and *ZNF304* have not been previously reported in CRC. *AKR1B1*, aldo-keto reductase family 1, member B1, catalyzes the reduction of aldehydes including the aldehyde form of glucose. It was reported to be downregulated in endometrial cancer and gastric cancer [37,38]. The product of *CHST10*, carbohydrate sulfotransferase 10, is known to inhibit the invasiveness of melanoma cells [39]. *ELOVL4* gene product, elongation of very long chain fatty acids 4, is responsible for the biosynthesis of fatty acids [40]. Hypermethylation of *ELOVL4* was reported in hepatocellular carcinoma and pancreatic adenocarcinoma by genome-wide methylation analysis [41,42]. *SOX5* is a member of the *SOX* (SRY-related HMG-box) family of transcription factors. It is well known that SOX5 regulates embryonic development and determines cell fate [43]. *STK33*, serine-threonine kinase 33, is located on chromosome 11p15.3, a gene-rich region that has been associated with human diseases and malignancies [44]. *ZNF304*, zinc finger protein 304, combines two conserved domains, class II AU-rich elements and a Krüppel-associated box, and is associated with the regulation of lymphocyte activation [45].

DNA methylation-mediated silencing of gene expression can be restored by demethylation agents such as 5-aza-dC. DNA methyltransferase inhibitor, 5′-azacytidine, may act as an inducer of cell differentiation by causing de-methylation and re-expression of genes silenced by hypermethylation [46]. 5-Azacytidine was approved in 2004 by the US Food and Drug Administration for treating myelodysplastic syndrome [47], and 5-aza-dC as a 5-azacytidine analog was widely used in DNA methylation studies [48]. Vincristine is a microtubule inhibitor and is commonly used for chemotherapy in pediatric acute lymphoblastic patients [32]. Several anticancer drugs are associated with drug-induced DNA hypermethylation in human lung adenocarcinoma and rhabdomyosarcoma cells [33]. Interestingly, the methylated cytosine was reduced after treatment with concentration of vincristine less than 100 μmole but it was induced after treatment with higher than 1000 μmole in human lung adenocarcinoma cells [33]. In this study, we selected 100 nM as an optimal concentration of vincristine which does not effect on the viability of CRC cells using MTT assay. Vincristine induced demethylation of methylated genes in CRC cells to the same extent as 5-aza-dC. In addition, vincristine restored the mRNA expression of *CHST10*, *ELOVL4*, *FLI1*, *STK33*, *SOX5*, and *ZNF304* in CRC cells. Interestingly, the methylation status of *AKR1B1* was not affected, but its mRNA expression was increased by both drugs. It may be regulated by upstream genes, with a demethylating effect by both drugs. Our results provide insights into the potential functional impact of vincristine on methylated genes in CRC.

## Conclusions

This study has identified novel candidate genes, *AKR1B1*, *CHST10*, *ELOVL4*, *SOX5*, *STK33*, and *ZNF304*, and provided evidence for their suitability as methylation biomarkers of CRC. We also analyzed the DNA methylation-based therapeutic effects of vincristine in CRC.

## Abbreviations

ACTB: Beta-actin; ADAMTS1: A disintegrin and metalloproteinase with thrombospondin motifs 1; ADHFE1: Alcohol dehydrogenase, iron containing, 1; AKR1B1: Aldo-keto reductase family 1; APC: Antigen-presenting cell; BEND4: BEN domain containing 4; BTG4: B-cell translocation gene 4; CACNA1G: Calcium, voltage-dependent, T type, alpha 1G subunit; CDKN2A: Cyclin-dependent kinase inhibitor 2A; CHFR: Checkpoint with forkhead and ring finger domains; CHST10: Carbohydrate sulfotransferase 10; CIMP: CpG island methylator phenotype; CNRIP1: Cannabinoid receptor interacting protein 1; COP: Cyclophosphamide, vincristine and prednisone; CRABP1: Cellular retinoic acid-binding protein 1; CRC: Colorectal cancer; DAPK1: Death-associated protein kinase 1; ELOVL4: Elongation of very long chain fatty acids protein 4; EYA4: Eyes absent homolog 4; FDR: False discovery rate; FLI1: Friend leukemia integration 1 transcription factor; GLP1R: Glucagon-like peptide 1 receptor; IGFBP3: Insulin-like growth factor-binding protein 3; IGF2: Insulin-like growth factor 2; IRF4: Interferon regulatory factor 4; JAM2: Junctional adhesion molecule B; MGMT: O-6-methylguanine-DNA methyltransferase; MLH1: MutL homolog 1; M-MLV: Moloney murine leukemia virus; MTT: 3-(4,5-dimethylthiazol-2-yl)-2,5-diphenyltetrazolium; NEUROG1: Neurogenin-1; NPTX1: Neuronal pentraxin I; NRG1: Neuregulin 1; QKI: Quaking homolog, KH domain RNA binding; QMSP: Quantitative methylation-specific polymerase chain reaction; PMR: Percentage of methylated reference; RASSF1A: Ras association domain-containing protein 1; RT-PCR: Reverse transcription polymerase chain reaction; RUNX3: Runt-related transcription factor 3; SLC8A3: Solute carrier family 8, member 3; SFRP1: Secreted frizzled-related protein 1; SFRP2: Secreted frizzled-related protein 1; SOX5: SPY(sex determining region Y)-box 5; SPG20: Spastic paraplegia 20; STK33: Serine-threonine kinase 33; ST6GALNAC5: ST6 (alpha-N-acetyl-neuraminyl-2,3-beta-galactosyl-1,3)-N-acetylgalactosaminide alpha-2,6-sialyltransferase 5; TAC1: Protachykinin-1; THBD: Thrombomodulin; THBS: Thrombospondin; TWIST1: Twist-related protein 1; UNC5C: Unc-5 homolog C; VSX2: Visual system homeobox 2; WRN: Werner syndrome; ZNF272: Zinc finger protein 272; ZNF304: Zinc finger protein 304; 5-aza-dC: 5-aza-2′-deoxycytidine.

## Competing interests

The authors declare that they have no competing interests.

## Authors’ contributions

JWM designed the study and drafted the manuscript. SKL and YWL prepared the clinical specimens and participated in the organization of clinical data. Dr. JOL, NK and SJK participated in the analysis of methylation array results and searching references. Dr. HJK helped the arrangement of results. Prof. JK provided the clinical specimens and clinicopathologic informations. Prof. HSK helped analyzed the results and revised the manuscript. Prof. SHP supervised the study and revised the manuscript. All authors read and approved the final manuscript.

## Supplementary Material

Additional file 1: Table S1 Primers for Quantitative methylation specific PCR (QMSP). **Table S2.** Primers for mRNA expression.
Click here for file

Additional file 2: Figure S1 Cell viability of CCD18Co, SW480, DLD-1, and LoVo cells by 5-aza-dC and vincristine. Cell viability of four cell lines after treatment with various concentrations of 5-aza-dC and vincristine for three days was evaluated by MTT assay. The cell viability of four cell lines was significantly reduced after treatment with concentration of 5-aza-dC greater than 30 μM **(A)** and did not change significantly after treatment with concentrations of vincristine less than 100 nM for two or three days **(B)**. *p-Values of <0.05 were considered statistically significant. Click here for file

## References

[B1] Grady WM Carethers JM Genomic and epigenetic instability in colorectal cancer pathogenesis Gastroenterology 2008 135 1079 1099 10.1053/j.gastro.2008.07.076 18773902 PMC2866182

[B2] Kim MS Lee J Sidransky D DNA methylation markers in colorectal cancer Cancer Metastasis Rev 2010 29 181 206 10.1007/s10555-010-9207-6 20135198

[B3] Dotan E Cohen SJ Challenges in the management of stage II colon cancer Semin Oncol 2011 38 511 520 10.1053/j.seminoncol.2011.05.005 21810510 PMC3242408

[B4] Alberts SR Sargent DJ Nair S Mahoney MR Mooney M Thibodeau SN Smyrk TC Sinicrope FA Chan E Gill S Effect of oxaliplatin, fluorouracil, and leucovorin with or without cetuximab on survival among patients with resected stage III colon cancer: a randomized trial JAMA 2012 307 1383 1393 10.1001/jama.2012.385 22474202 PMC3442260

[B5] Vogelstein B Fearon ER Hamilton SR Kern SE Preisinger AC Leppert M Nakamura Y White R Smits AM Bos JL Genetic alterations during colorectal-tumor development N Engl J Med 1988 319 525 532 10.1056/NEJM198809013190901 2841597

[B6] Lao VV Grady WM Epigenetics and colorectal cancer Nat Rev Gastroenterol Hepatol 2011 8 686 700 10.1038/nrgastro.2011.173 22009203 PMC3391545

[B7] Pogribny IP Beland FA DNA methylome alterations in chemical carcinogenesis Cancer Lett 2013 334 39 45 10.1016/j.canlet.2012.09.010 23010082

[B8] Kondo Y Issa JP Epigenetic changes in colorectal cancer Cancer Metastasis Rev 2004 23 29 39 15000147 10.1023/a:1025806911782

[B9] Hinoue T Weisenberger DJ Lange CP Shen H Byun HM Van Den Berg D Malik S Pan F Noushmehr H van Dijk CM Genome-scale analysis of aberrant DNA methylation in colorectal cancer Genome Res 2012 22 271 282 10.1101/gr.117523.110 21659424 PMC3266034

[B10] Baylin SB Herman JG DNA hypermethylation in tumorigenesis: epigenetics joins genetics Trends Genet 2000 16 168 174 10.1016/S0168-9525(99)01971-X 10729832

[B11] Rashid A Shen L Morris JS Issa JP Hamilton SR CpG island methylation in colorectal adenomas Am J Pathol 2001 159 1129 1135 10.1016/S0002-9440(10)61789-0 11549606 PMC1850474

[B12] Suzuki H Watkins DN Jair KW Schuebel KE Markowitz SD Chen WD Pretlow TP Yang B Akiyama Y Van Engeland M Epigenetic inactivation of SFRP genes allows constitutive WNT signaling in colorectal cancer Nat Genet 2004 36 417 422 10.1038/ng1330 15034581

[B13] Rodriguez J Frigola J Vendrell E Risques RA Fraga MF Morales C Moreno V Esteller M Capella G Ribas M Peinado MA Chromosomal instability correlates with genome-wide DNA demethylation in human primary colorectal cancers Cancer Res 2006 66 8462 9468 10.1158/0008-5472.CAN-06-0293 16951157

[B14] Weisenberger DJ Siegmund KD Campan M Young J Long TI Faasse MA Kang GH Widschwendter M Weener D Buchanan D CpG island methylator phenotype underlies sporadic microsatellite instability and is tightly associated with BRAF mutation in colorectal cancer Nat Genet 2006 38 787 793 10.1038/ng1834 16804544

[B15] Ogino S Cantor M Kawasaki T Brahmandam M Kirkner GJ Weisenberger DJ Campan M Laird PW Loda M Fuchs CS CpG island methylator phenotype (CIMP) of colorectal cancer is best characterised by quantitative DNA methylation analysis and prospective cohort studies Gut 2006 55 1000 1006 10.1136/gut.2005.082933 16407376 PMC1856352

[B16] Noffsinger AE Serrated polyps and colorectal cancer: new pathway to malignancy Annu Rev Pathol 2009 4 343 364 10.1146/annurev.pathol.4.110807.092317 19400693

[B17] Snover DC Update on the serrated pathway to colorectal carcinoma Hum Pathol 2011 42 1 10 10.1016/j.humpath.2010.06.002 20869746

[B18] Dahlin AM Palmqvist R Henriksson ML Jacobsson M Eklof V Rutegard J Oberg A Van Guelpen BR The role of the CpG island methylator phenotype in colorectal cancer prognosis depends on microsatellite instability screening status Clin Cancer Res 2010 16 1845 1855 10.1158/1078-0432.CCR-09-2594 20197478

[B19] Ogino S Nosho K Kirkner GJ Kawasaki T Meyerhardt JA Loda M Giovannucci EL Fuchs CS CpG island methylator phenotype, microsatellite instability, BRAF mutation and clinical outcome in colon cancer Gut 2009 58 90 96 10.1136/gut.2008.155473 18832519 PMC2679586

[B20] Ogino S Kawasaki T Kirkner GJ Kraft P Loda M Fuchs CS Evaluation of markers for CpG island methylator phenotype (CIMP) in colorectal cancer by a large population-based sample J Mol Diagn 2007 9 305 314 10.2353/jmoldx.2007.060170 17591929 PMC1899428

[B21] Hughes LA Khalid-de Bakker CA Smits KM Van den Brandt PA Jonkers D Ahuja N Herman JG Weijenberg MP Van Engeland M The CpG island methylator phenotype in colorectal cancer: progress and problems Biochim Biophys Acta 2012 1825 77 85 22056543 10.1016/j.bbcan.2011.10.005

[B22] Oster B Thorsen K Lamy P Wojdacz TK Hansen LL Birkenkamp-Demtroder K Sorensen KD Laurberg S Orntoft TF Andersen CL Identification and validation of highly frequent CpG island hypermethylation in colorectal adenomas and carcinomas Int J Cancer 2011 129 2855 2866 10.1002/ijc.25951 21400501

[B23] Kim YH Lee HC Kim SY Yeom YI Ryu KJ Min BH Kim DH Son HJ Rhee PL Kim JJ Epigenomic analysis of aberrantly methylated genes in colorectal cancer identifies genes commonly affected by epigenetic alterations Ann Surg Oncol 2011 18 2338 2347 10.1245/s10434-011-1573-y 21298349 PMC3393129

[B24] Mori Y Olaru AV Cheng Y Agarwal R Yang J Luvsanjav D Yu W Selaru FM Hutfless S Lazarev M Novel candidate colorectal cancer biomarkers identified by methylation microarray-based scanning Endocr Relat Cancer 2011 18 465 478 10.1530/ERC-11-0083 21636702 PMC3464012

[B25] Eads CA Danenberg KD Kawakami K Saltz LB Blake C Shibata D Danenberg PV Laird PW MethyLight: a high-throughput assay to measure DNA methylation Nucleic Acids Res 2000 28 E32 10.1093/nar/28.8.e32 10734209 PMC102836

[B26] Tobin WE Sandler G Depression of muscle spindle function with vincristine Nature 1966 212 90 91 10.1038/212090a0 5965582

[B27] Yahalom J Varsos G Fuks Z Myers J Clarkson BD Straus DJ Adjuvant cyclophosphamide, doxorubicin, vincristine, and prednisone chemotherapy after radiation therapy in stage I low-grade and intermediate-grade non-Hodgkin lymphoma. Results of a prospective randomized study Cancer 1993 71 2342 2350 10.1002/1097-0142(19930401)71:7<2342::AID-CNCR2820710728>3.0.CO;2-I 8453557

[B28] Gingrich RD Armitage JO Burns CP Treatment of adult acute lymphoblastic leukemia with cytosine arabinoside, vincristine, and prednisone Cancer Treat Rep 1978 62 1389 1391 356988

[B29] Kolarić K Roth A Vukas D Combination chemotherapy with adriamycin, cyclophosphamide, methotrexate and vincristine in lung cancer patients with extensive disease Tumori 1979 65 635 642 229601 10.1177/030089167906500512

[B30] Taylor CW Dalton WS Mosley K Dorr RT Salmon SE Combination chemotherapy with cyclophosphamide, vincristine, adriamycin, and dexamethasone (CVAD) plus oral quinine and verapamil in patients with advanced breast cancer Breast Cancer Res Treat 1997 42 7 14 10.1023/A:1005716214718 9116320

[B31] Fleischer I Wainstein R de Gibson AS Treatment of advanced cancer of the colon and rectum with the combination of 5-fluorouracil, imidazolecarboxamide and vincristine Medicina (B Aires) 1983 43 143 146 6664281

[B32] Tang TC Kuo MC Chang H Dunn P Wang PN Wu JH Lin TL Hung YS Kuo TT Shih LY Primary colonic lymphoma: an analysis of 74 cases with localized large-cell lymphoma Eur J Haematol 2011 87 28 36 10.1111/j.1600-0609.2011.01632.x 21535155

[B33] Nyce J Drug-induced DNA hypermethylation and drug resistance in human tumors Can Res 1989 49 5829 5836 2790794

[B34] Maruyama R Sugio K Yoshino I Maehara Y Gazdar AF Hypermethylation of FHIT as a prognostic marker in nonsmall cell lung carcinoma Cancer 2004 100 1472 1477 10.1002/cncr.20144 15042681

[B35] Uhm KO Lee ES Lee YM Kim HS Park YN Park SH Aberrant promoter CpG islands methylation of tumor suppressor genes in cholangiocarcinoma Oncol Res 2008 17 151 157 10.3727/096504008785114110 18773859

[B36] Uhm KO Lee JO Lee YM Lee ES Kim HS Park SH Aberrant DNA methylation of integrin alpha4: a potential novel role for metastasis of cholangiocarcinoma J Cancer Res Clin Oncol 2010 136 187 194 10.1007/s00432-009-0646-9 19655168 PMC11827953

[B37] Hevir N Sinkovec J Lanisnik Rizner T Decreased levels of AKR1B1 and AKR1B10 in cancerous endometrium compared to adjacent non-cancerous tissue Chem Biol Interact 2013 202 226 233 10.1016/j.cbi.2012.11.001 23146748

[B38] Kropotova ES Zinov'eva OL Zyrianova AF Choinzonov EL Afanas'ev SG Cherdyntseva NV Beresten SF Oparina N Mashkova TD Expression of genes involved in retinoic acid biosynthesis in human gastric cancer Mol Biol (Mosk) 2013 47 317 330 23808167 10.7868/s0026898413020079

[B39] Zhao X Graves C Ames SJ Fisher DE Spanjaard RA Mechanism of regulation and suppression of melanoma invasiveness by novel retinoic acid receptor-gamma target gene carbohydrate sulfotransferase 10 Can Res 2009 69 5218 5225 10.1158/0008-5472.CAN-09-0705 19470764

[B40] Zhang K Kniazeva M Han M Li W Yu Z Yang Z Li Y Metzker ML Allikmets R Zack DJ A 5-bp deletion in ELOVL4 is associated with two related forms of autosomal dominant macular dystrophy Nat Genet 2001 27 89 93 11138005 10.1038/83817

[B41] Revill K Wang T Lachenmayer A Kojima K Harrington A Li J Hoshida Y Llovet JM Powers S Genome-Wide Methylation Analysis and Epigenetic Unmasking Identify Tumor Suppressor Genes in Hepatocellular Carcinoma Gastroenterology 2013 145 1424 1435 10.1053/j.gastro.2013.08.055 24012984 PMC3892430

[B42] Omura N Li CP Li A Hong SM Walter K Jimeno A Hidalgo M Goggins M Genome-wide profiling of methylated promoters in pancreatic adenocarcinoma Cancer Biol Ther 2008 7 1146 1156 10.4161/cbt.7.7.6208 18535405 PMC2763640

[B43] Lefebvre V The SoxD transcription factors–Sox5, Sox6, and Sox13–are key cell fate modulators Int J Biochem Cell Biol 2010 42 429 432 10.1016/j.biocel.2009.07.016 19647094 PMC2826538

[B44] Nowak NJ Shows TB Genetics of chromosome 11: loci for pediatric and adult malignancies, developmental disorders, and other diseases Cancer Invest 1995 13 646 659 10.3109/07357909509024936 7583717

[B45] Sabater L Ashhab Y Caro P Kolkowski EC Pujol-Borrell R Dominguez O Identification of a KRAB-containing zinc finger protein, ZNF304, by AU-motif-directed display method and initial characterization in lymphocyte activation Biochem Biophys Res Commun 2002 293 1066 1072 10.1016/S0006-291X(02)00344-3 12051768

[B46] Kaminskas E Farrell AT Wang YC Sridhara R Pazdur R FDA drug approval summary: azacitidine (5-azacytidine, Vidaza) for injectable suspension Oncologist 2005 10 176 182 10.1634/theoncologist.10-3-176 15793220

[B47] Peedicayil J Epigenetic therapy–a new development in pharmacology Indian J Med Res 2006 123 17 24 16567863

[B48] Christman JK 5-Azacytidine and 5-aza-2′-deoxycytidine as inhibitors of DNA methylation: mechanistic studies and their implications for cancer therapy Oncogene 2002 21 5483 5495 10.1038/sj.onc.1205699 12154409

