# Supplementary material for: Identification of novel hypermethylated genes and demethylating effect of vincristine in colorectal cancer
Source: J Exp Clin Cancer Res. 2014 Jan 6;33(1):4. doi: 10.1186/1756-9966-33-4 (PMC3923411; doi:10.1186/1756-9966-33-4)
Supplement: Additional file 1: Table S1 — Primers for Quantitative methylation specific PCR (QMSP). Table S2. Primers for mRNA expression. [file 1756-9966-33-4-S1.doc]

**Supplementary Table 1.** Primers for Quantitative methylation specific PCR (QMSP)

| **Categories** | **Genes** | **Primer sequences (5'🡪3')** | | **Location** | **Product sizes (bp)** | **Annealing (℃)** | **Gene bank** |
| --- | --- | --- | --- | --- | --- | --- | --- |
| **candidate**  **genes**  **(21)** | ADHFE1 | F ; | AGGGCGGTATTTAAATTTTTCGAATT | (-112~+190) | 302 | 60 | NC_000008.10 |
|  |  | R ; | CGCGAAACGAATAAACAAACGCGACCGA |  |  |  |  |
|  | AKR1B1 | F ; | CGGAAGAAGTATTTTCGTCGA | (-120~+45) | 166 | 60 | NC_000007.13 |
|  |  | R ; | CAATACGATACGACCTTAACCG |  |  |  |  |
|  | BTG4 | F ; | GTATAATACGCGTAGTTGGGTTAGC | (-422~-252) | 171 | 60 | NC_000011.9 |
|  |  | R ; | AAAAAAACGAAAAAAACCTAAACG |  |  |  |  |
|  | CHST10 | F ; | TTTTTGTAGCGGTAGAAAGGGAGATTCG | (-125~+72) | 198 | 58 | NC_000002.11 |
|  |  | R ; | GACTTTAAAAACCAAAACGCCGAC |  |  |  |  |
|  | COL4A1 | F ; | TTCGGCGTATTGTAGTTATATTTTC | (-156~-22) | 135 | 60 | NC_000013.10 |
|  |  | R ; | GAACTCCCCAATTTATTAACGCT |  |  |  |  |
|  | ELOVL4 | F ; | GAGTTTAGGTGTTTCGTTTTCGTTC | (-158~-42) | 114 | 56 | NC_000006.11 |
|  |  | R ; | CCTCCCTCCCTAATATTAAAACTCG |  |  |  |  |
|  | EYA4 | F ; | GTTATTCGAGGTTAAATAAAAACGG | (-198~-50) | 149 | 60 | NC_000006.11 |
|  |  | R ; | ACTTACGCAAAAAAATAAAACGAA |  |  |  |  |
|  | FLI1 | F ; | TTCGTTTCGTTATAATAATAAACGT | (-108~+197) | 305 | 52 | NC_000011.9 |
|  |  | R ; | AAAAAAAACCACGTCTTCCG |  |  |  |  |
|  | HCN1 | F ; | GAGTTAGGCGCGTAGTTAGTAGC | (-163~-42) | 122 | 60 | NC_000005.9 |
|  |  | R ; | TAACCGAAAAAATAAAAACCCG |  |  |  |  |
|  | LRRC4 | F ; | ATCGTAGGTTTTTTTAGAGAGTTCGG | (-743~-1047) | 304 | 60 | NC_000007.13 |
|  |  | R ; | CCGCTACTACTACTACCGCCGCT |  |  |  |  |
|  | NDRG4 | F ; | GTATTTTAGTCGCGTAGAAGGC | (-120~-9) | 112 | 60 | NC_000016.9 |
|  |  | R ; | AATTTAACGAATATAAACGCTCGAC |  |  |  |  |
|  | QKI | F ; | GATTTAGTTTTTAGTCGGTTGTCGT | (-734~-535) | 200 | 60 | NC_000006.11 |
|  |  | R ; | AAACTTTCGAAATAAACATCACGTA |  |  |  |  |
|  | SDC2 | F ; | TCGAATTTTTAAGGTAGAAAAGTTATATAC | (-317~-73) | 245 | 60 | NC_000008.10 |
|  |  | R ; | AACTCCGAAAACCAATAAACG |  |  |  |  |
|  | SLC8A3 | F ; | TTTTTGAGGATAAGGGGAAAGTC | (-311~+26) | 338 | 60 | NC_000014.8 |
|  |  | R ; | ACCCTATCAACTAAAAAACAACGC |  |  |  |  |
|  | SOX5 | F ; | TTTTTGTACGGTGTGGGTAATCGTC | (-134~-511) | 377 | 62 | NC_000012.11 |
|  |  | R ; | AAATCCCTTTACTAATCATAAAACGAC |  |  |  |  |
|  | SPG20 | F ; | GTCGAGTAGTCGACGTGGTC | (-246~-78) | 168 | 60 | NC_000013.10 |
|  |  | R ; | AATAATACGTAAAAAAACGTCCGTC |  |  |  |  |
|  | STK33 | F ; | GTGCGTATTTGTCGGAGATTC | (-164~-35) | 130 | 55 | NC_000011.9 |
|  |  | R ; | TACCATAACAACGACCTAACCG |  |  |  |  |
|  | TFPI2 | F ; | GTTTCGTTTTGATTTAAGAATTTCG | (-141~-19) | 123 | 60 | NC_000007.13 |
|  |  | R ; | CGAAAATAACCTACTAATAATTACGCT |  |  |  |  |
|  | UNC5C | F ; | GTTTAGGTTTGGCGTATCGC | (-463~-245) | 219 | 60 | NC_000004.11 |
|  |  | R ; | GCCAAAAAAACGTAAAAAACG |  |  |  |  |
|  | ZNF272 | F ; | TAGAAGGGATTTTTCGATTTTTCGA | (-290~-168) | 123 | 60 | NC_000019.9 |
|  |  | R ; | ACCGAATATAAAAACTCACCCAACG |  |  |  |  |
|  | ZNF304 | F ; | TTTGGTAGATATTCGGGAGAATTC | (-194~+16) | 211 | 60 | NC_000019.9 |
|  |  | R ; | GAACCGAACATAAAAAAAATACGTC |  |  |  |  |
| **CIMP**  **Markers**  **(18)** | ADAMTS1 | F ; | TAATTGAGTTATCGTAATCGGGC | (-341~-183) | 159 | 65 | NC_000021.8 |
|  |  | R ; | CACTACTCGTCAATCTAAAAACGAA |  |  |  |  |
|  | APC | F ; | TATTGCGGAGTGCGGGTC | (-135~-37) | 98 | 60 | NC_000005.9 |
|  |  | R ; | TCGACGAACTCCCGACGA |  |  |  |  |
|  | CACNA1G | F ; | TTTCGGAGTTCGCGTTGTTCGGGTTT | (-532~-360) | 173 | 65 | NC_000017.10 |
|  |  | R ; | CGAATCGCCCGAAACCCCGACTA |  |  |  |  |
|  | CHFR | F ; | TAATTGTATTCGAAAGGGTTTTTAC | (-347~-131) | 217 | 65 | NC_000012.11 |
|  |  | R ; | TTAATCCTAACCAAACGACTTCG |  |  |  |  |
|  | CRABP1 | F ; | TTGAGCGTATATCGAGTAGGGGTTGC | (-98~+39) | 137 | 58 | NC_000015.9 |
|  |  | R ; | ACGAAAACTAACGCTTTACGC |  |  |  |  |
|  | DAPK1 | F ; | TATATATTTCGGGACGGAAGAATC | (-318~-28) | 291 | 65 | NC_000009.11 |
|  |  | R ; | TAAAAACCCTACAAACGAACTAACG |  |  |  |  |
|  | IGF2 | F ; | CGTTTGTTCGCGTTTTGTTTATTAGC | (-336~-141) | 196 | 60 | NC_000011.9 |
|  |  | R ; | ACCGCCTCCTCGAACGAAACG |  |  |  |  |
|  | IGFBP3 | F ; | GTTTCGGGCGTGAGTACGA | (-215~-117) | 99 | 60 | NC_000007.13 |
|  |  | R ; | GAATCGACGCAAACACGACTAC |  |  |  |  |
|  | MGMT | F ; | GTTTGTATTGGTTGAAGGGTTATTT | (-292~-184) | 109 | 60 | NC_000010.10 |
|  |  | R ; | CTAAAACAATCTACACATCCTCACT |  |  |  |  |
|  | NEUROG1 | F ; | TATGTAAATATTCGGGCGTTGTAC | (-155~+43) | 199 | 60 | NC_000005.9 |
|  |  | R ; | GATCTCCTAAATAATATCGCCGAC |  |  |  |  |
|  | p16INK4a | F ; | ACGCGAGTAGTATTAGAATTCGC | (-370~-224) | 126 | 60 | NC_000009.11 |
|  |  | R ; | ATAACGACCCAAAAACCGAA |  |  |  |  |
|  | RASSF1A | F ; | TTTTGTATTTAGGTTTTTATTGCGC | (-158~-13) | 146 | 60 | NC_000003.11 |
|  |  | R ; | CCGTACTTCGCTAACTTTAAACG |  |  |  |  |
|  | RUNX3 | F ; | TGTTCGCGATGGGGGTTTCGTCGATTG | (-102~+64) | 166 | 60 | NC_000001.10 |
|  |  | R ; | CGAAACTCGCCCGCGACCGCCCCGACTC |  |  |  |  |
|  | SFRP1 | F ; | GAATTCGTTCGCGAGGGA | (-128~-59) | 70 | 60 | NC_000008.10 |
|  |  | R ; | AAACGAACCGCACTCGTTACC |  |  |  |  |
|  | SFRP2 | F ; | GCGTTTTAGTCGTCGGTTGTTAGT | (-143~-76) | 68 | 60 | NC_000004.11 |
|  |  | R ; | AAACGACCGAAATTCGAACTTATC |  |  |  |  |
|  | TAC1 | F ; | TTAGATTTGTAGACGGAAGTAGGTC | (-135~+3) | 139 | 60 | NC_000007.13 |
|  |  | R ; | GTAATTAAAAATTTCCGAAACGAT |  |  |  |  |
|  | THBD | F ; | GGGCGGTTAGAGAATTTAGTAATTC | (-136~+13) | 150 | 65 | NC_000020.10 |
|  |  | R ; | CTACGAAACAACCTCTAACATACGA |  |  |  |  |
|  | WRN | F ; | TTGAGAATAATCGTAGACGTTTTTC | (-472~-176) | 252 | 65 | NC_000008.10 |
|  |  | R ; | AAAATAATATAAATACCCGCCGAC |  |  |  |  |
| **Control** | ACTB | F ; | TGGTGATGGAGGAGGTTTAGTAAGT | (-1645~-1513) | 132 | 60 | NC_000007.13 |
|  |  | R ; | AACCAATAAAACCTACTCCTCCCTTAA |  |  |  |  |

**Supplementary Table 2.** Primers for mRNA expression

| **Genes** | **Primer sequences (5'à3')** | | **Product sizes (bp)** | **Annealing (℃)** | **Gene bank** |
| --- | --- | --- | --- | --- | --- |
| AKR1B1 | F ; | CCCATGTGTACCAGAATGAGAA | 362 | 60 | NM_001628 |
|  | R ; | CTGGAGATGGTTGAAGTTGGAG |  |  |  |
| CHST10 | F ; | GTGTGATTGGACACCACGAG | 176 | 60 | NM_004854.4 |
|  | R ; | ATACAGGCGTCGGATGTCTC |  |  |  |
| ELOVL4 | F ; | TAAGTGGGTTGCAGGAGGAC | 212 | 60 | NM_022726.3 |
|  | R ; | GGAAGGGGCAGTCAGTGTAA |  |  |  |
| EYA4 | F ; | CTGCATAAATGTCTTGGTAACGAC | 260 | 60 | NM_004100 |
|  | R ; | GGAGGTCTGAGTGACTGGATATCC |  |  |  |
| FLI1 | F ; | CAACAGCTATATGGACGAGAAGAA | 356 | 60 | NM_001167681 |
|  | R ; | AAGGGTCTTCTTTGACACTCAATC |  |  |  |
| SOX5 | F ; | GTGGCCATAGGACTCCCACT | 134 | 64 | NM_006940.4 |
|  | R ; | ACTCTGTCGCCCACCTTCTT |  |  |  |
| STK33 | F ; | CTTCGGTGAGACCAACCAAT | 159 | 60 | NM_030906.2 |
|  | R ; | TGTAATTGGCATCAGGGACA |  |  |  |
| ZNF304 | F ; | CACTTGTGCAACACCAAAAA | 182 | 60 | NM_020657.2 |
|  | R ; | GTGCAAAATGAGGCTGGAGT |  |  |  |
| ACTB | F ; | AGAGCTACGAGCTGCCTGAC | 184 | 60 | NM_001101.3 |
|  | R ; | AGCACTGTGTTGGCGTACAG |  |  |  |
