# Supplementary figures and images for: Identification of novel hypermethylated genes and demethylating effect of vincristine in colorectal cancer
Source: J Exp Clin Cancer Res. 2014 Jan 6;33(1):4. doi: 10.1186/1756-9966-33-4 (PMC3923411; doi:10.1186/1756-9966-33-4)

## Slide 1
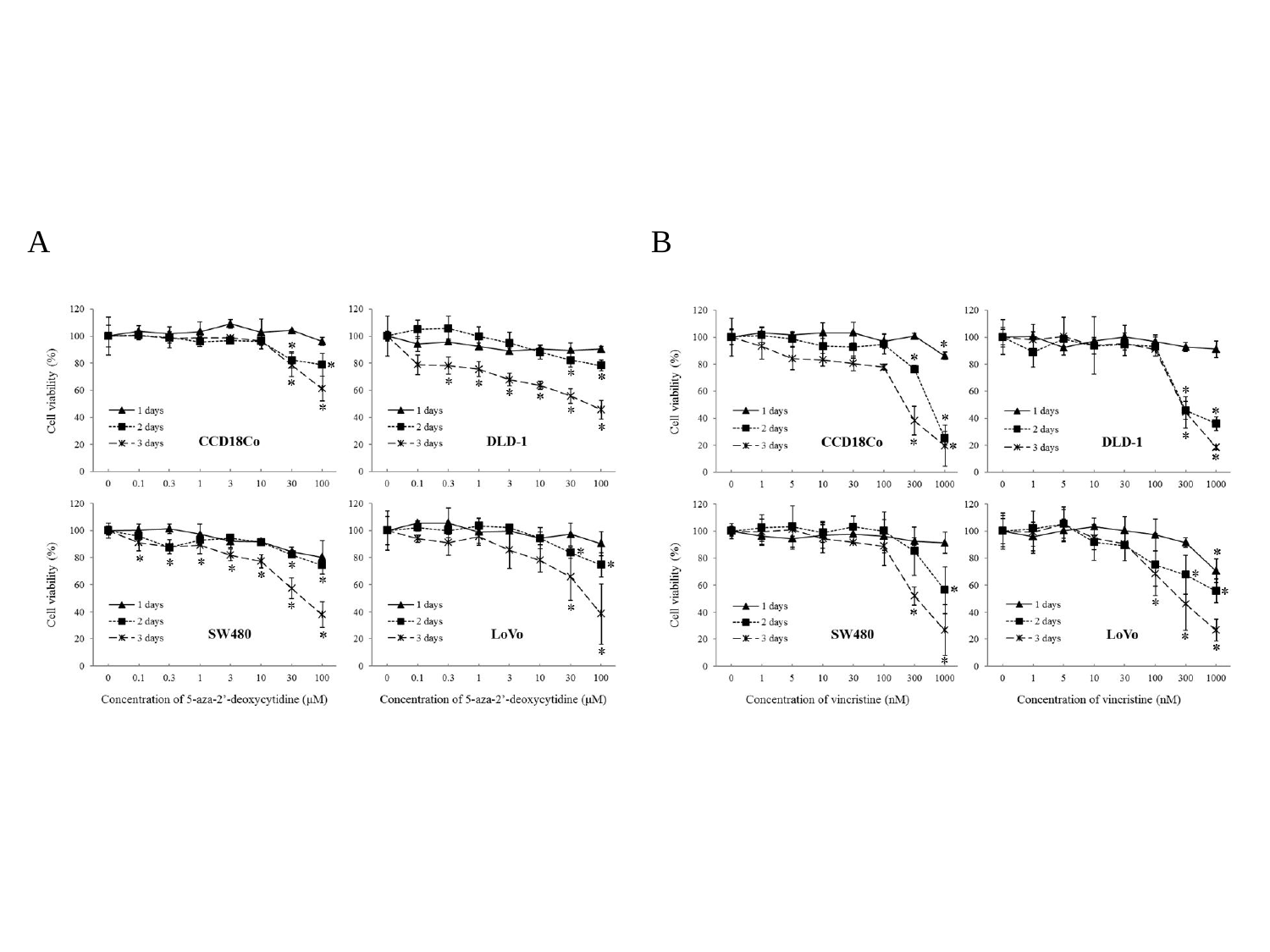

A
B

Supplement: Additional file 2: Figure S1 — Cell viability of CCD18Co, SW480, DLD-1, and LoVo cells by 5-aza-dC and vincristine. Cell viability of four cell lines after treatment with various concentrations of 5-aza-dC and vincristine for three days was evaluated by MTT assay. The cell viability of four cell lines was significantly reduced after treatment with concentration of 5-aza-dC greater than 30 μM (A) and did not change significantly after treatment with concentrations of vincristine less than 100 nM for two or three days (B). *p-Values of <0.05 were considered statistically significant. [file 1756-9966-33-4-S2.pptx]
